# Sharp transitions of gamma coherence in inhibitory networks occur when a biological context and constraints are imposed

**DOI:** 10.1186/1471-2202-14-S1-P365

**Published:** 2013-07-08

**Authors:** Katie A Ferguson, Carey YL Huh, Bénédicte Amilhon, Sylvain Williams, Frances K Skinner

**Affiliations:** 1 Toronto Western Research Institute, University Health Network, Toronto, Ontario, M5T 2S8, Canada; 2 Physiology, University of Toronto, Toronto, Ontario, M5S 1A1, Canada; 3 Medicine (Neurology), Physiology, Institute of Biomaterials and Biomedical Engineering, University of Toronto, Toronto, Ontario, M5S 1A1, Canada; 4 Psychiatry, Douglas Mental Health University Institute, McGill University, Montreal, Quebec, H4G 1X6, Canada

## 

From several experimental and modeling studies, it has become apparent that networks of parvalbumin-positive (PV+), fast-firing interneurons play an essential role in generating population gamma rhythms [1]. Many features of these perisomatically-inhibiting PV+ cell networks influence the generation of gamma oscillations, a number of which have been explored in network models [2,3]. These include the synaptic delay, the decay time constant of the postsynaptic conductance, and whether the reversal potential is hyperpolarizing or shunting. While it is clear that all these features affect whether and how much coherence occurs at gamma frequencies, it is much less clear which features (i.e. parameters and parameter balances) may be essential in various biological contexts. To address this, we used our previously constructed network model of hippocampal CA1 PV+ cells [4]. Network size, connectivity, and cellular components would all be expected to affect the network dynamics. Thus, experimental data from an intact hippocampus *in vitro *was used to obtain a clear biological context, and importantly, the amount of input that these cells receive during ongoing theta population activities was estimated. Using these cellular components and input characteristics, we found that coherent gamma oscillations could emerge within experimental constraints for particular parameter balances. Here we investigate the effect of appropriately sized and connected networks.

Each cell in the network received excitatory input (I_applied_), which was heterogeneous across cells, and synaptic inhibition from presynaptic interneurons, which had a particular maximal conductance value (g_PV_). We systematically varied g_PV _and I_applied _within the experimentally determined ranges, and determined the coherence of the network model population firing and the network frequency for each combination of these values. For each of these network simulation sets, we also varied the connectivity probability as well as network size to explore how these factors affect the network's ability to produce coherent network firing. We find that when our connection probability is similar to what has been found experimentally, our networks exhibit a sharp transition from random firing to network coherence with only a small change in I_applied_. However, as connectivity in the network is increased beyond experimentally estimated values, this sharp transition disappears. Instead, a larger window of coherence is achieved, but with a smooth transition from random to coherent firing. Similarly, decreasing the network size increases the window of coherent firing, and while these sharp transitions remain for changing network sizes, the parameter regimes in which they occur changes significantly when the network size becomes much smaller than experimental estimates. Our work indicates that CA1 fast-spiking PV+ networks are capable of producing gamma population rhythms, and that perturbation in and out of coherent states can occur abruptly given the sharp transitions obtained in our network simulations. Since our simulations approximate a biological context, it may be that gating in and out of coherence is a design property to allow generation of theta/gamma network oscillations.
